# Functionality and Interfaces of a Herd Health Decision Support System for Practising Dairy Cattle Veterinarians in New Zealand

**DOI:** 10.3389/fvets.2018.00021

**Published:** 2018-02-23

**Authors:** John I. Alawneh, Joerg Henning, Timothy W. J. Olchowy

**Affiliations:** ^1^School of Veterinary Science, University of Queensland, Gatton, QLD, Australia

**Keywords:** dairy, productivity, decision support systems, herd health, technology

## Abstract

Decision-making processes to assess and improve the health of dairy herds are often unstructured due to the complexity of interactions that exist between the health and productivity of the herd, for which there are no ready to hand solutions. Decisions made in the face of these complex herd health problems are often based on the experience and perceptions of what might be a quick or the easiest solution. To shift from this unstructured process to semistructured decision-making requires a more holistic understanding of potential health problems and access to herd productivity information and to analytical methods suitable for examining and evaluating such data. Technological advances in agriculture have made the development of such information technology systems both possible and relatively accessible to decision makers working with dairy herds (e.g., veterinarians). The timely access and appropriate analysis of herd productivity data provides the herd health advisor with the opportunity to track and benchmark the performance of dairy herds. Thus, a decision support system (DSS) will use best available evidence to guide the allocation of resources to specific, most promising herd health interventions. This article presents an example of a DSS-based on collection of data and algorithm of analysis.

## Introduction

Over the past 30 years, the New Zealand dairy industry has expanded with increases in cow numbers (2 million to over 5 million) and in the average size of milking herds (135 to over 400 cows). Concurrently, there has been a decrease in the number of dairy herds from 15,816 in 1982–1983 to 11,970 in 2015–2016 (1). Growing commercialization has affected several aspects of dairy farming including the management of herd health and productivity. Larger herd size requires the existence of a continuous and detailed decision-making and problem-solving process. Therefore, an efficient decision support system (DSS) framework is critical to enhance the work of farm management and health advisors (e.g., veterinarians) with their decision-making abilities focused on optimization of herd health and productivity.

Investment of dairy farmer into the state-of-art equipment, tools and software products have made it possible to monitor a range of outcomes including milk production parameters (e.g., bulk tank milk and individual cow milk yields and quality or the temperature of harvested milk), management parameters (e.g., herd structure, pasture availability), and environmental parameters (e.g., environmental temperature and rainfall) (2, 3). These technological advances have improved the ability of herd managers to recognize deviations of heard production parameters from the norm and to implement timely corrective interventions.

Assessment and decision-making processes about herd health are often unstructured due to the complexity of interactions and the lack of immediate solutions to health problems facing the herd. In the face of these complex herd problems, decisions made by busy veterinarians tend to be based on experience, trial and error, and ease of implementation. For example, a sudden increase in a herd’s bulk tank milk somatic cell count above the threshold set by the industry (400,000 cells/mL, New Zealand) downgrades the milk quality of that herd and could result in the dairy processor imposing significant financial penalties. This sudden change in milk quality is an indicator of an active mastitis outbreak in the milking herd. Farm management reacts by screening the milking herd to identify poor milk cows with poor milk quality and divert their milk from the milk supply.

A mastitis outbreak signals a potential flaw in the herd management’s mastitis quality control process. The outbreak might be caused by a shift in the proportion of cows “at risk” for mastitis, higher than normal exposure to mastitis causing pathogens, improper milking routine, process failure in mastitis prevention protocols, or a combination of these factors. Screening and isolating cows with high somatic cell counts (poor milk quality) is an example of the unstructured approach to decision-making, which only aims to temporarily contain the effect of the outbreak on milk quality and economic return. The creation of a structured, or a semi-structured, decision-making process to contain this outbreak requires a better understanding, monitoring, and evaluation of the different components of the herds’ health that could be influencing the risk of the outbreak. This in turn requires farm management to precisely define the set of relevant cow-level and herd-level milk quality information and to access any animal health database systems potentially critical to the decision-making process (4, 5). Milk quality and herd health information systems do exist, either on farm or online. However, accessing the appropriate information and deciding on the analytical methods to be used to derive an informed decision can be an overwhelming and challenging task for dairy veterinarian. An interactive and adaptable animal health DSS can help a dairy veterinarian in such decision-making. It should include analytical approaches appropriate for the identification of animal health problems and provide a range of solution options so that decision makers can effectively complete their decision-making processes and be able to predict consequences of interventions (4, 6).

In the context of the health of the herd, a well-designed DSS can be used to collect, store, and manage data. The data can be used for a number of purposes including descriptive and analytical epidemiological analyses. An effective DSS should have two main goals. The first goal is to provide herd health advisors with the ability to track both animal health and production performance. This is an essential requirement if farm managers and their advisors are to bench mark their herds against local and national herds. The second goal is to provide means to facilitate the identification of both the re-emergence of previous problems and development of new herd health problems. Prompt identification of a new or recurrent problem allows immediate and appropriate actions to deal with issues and to efficiently allocate the resources necessary to mitigate the negative impact of such a problem during subsequent production periods. A well-designed DSS system uses the best available evidence to develop interventions aimed at improving the herd health and productivity and supports the design of ongoing disease surveillance strategies.

In New Zealand, as a part of the quality assurance program of a dairy herd, the milk processors (dairy milk companies) record the milk quantity and quality (bulk tank milk somatic cell count and bulk milk fat and protein percentages) for each bulk milk collection. The Livestock Improvement Corporation (LIC), the main dairy farming co-operative that serves approximately 80% of dairy farms in New Zealand, holds information regarding the genetics of member herds. These data include individual cow milk quality (individual cow milk somatic cell count, milk fat and protein percentages, and production index), insemination records, pregnancy diagnosis records, and reproduction index data. Dairy farmers and their advisors can access this information to assist in making informed decisions with respect to the routine management of their herds.

A number of DSS exist (e.g., ALPRO™ herd management system, DeLaval Limited, Hamilton New Zealand; DairyMGT™, University of Wisconsin-Madison, USA) that are either for commercial use or time consuming for busy clinicians. Currently, reviewing and comparing available DSS is beyond the scope of this article. In this short paper, we outline the conceptual framework, the functionality and interfaces for an in-house, web-based herd health DSS designed for a large veterinary practice (number of veterinarians using the DSS = 8) in New Zealand (DSS*i*plus). DSS*i*plus was designed for veterinary advisors and was used to transform milk and reproductive performance data and accounting transaction records into useful information at low cost. The information is critical for evidence-based, herd health decision-making, evaluation of implanted herd health-focused interventions and herd benchmarking.

### Web-Based Herd Health Decision Support System: Concept and Methodology

Veterinarians are expected to assist farmers making complex decisions regarding maintaining good genetics of the herd and in the evaluation of the efficacy of mastitis and reproductive management strategies in the herd. The decisions are based on cows’ historic health records (e.g., clinical mastitis events or amount and type antibiotic use on farm) and reproductive events (e.g., insemination records). A careful evaluation of the efficacy of the herd mastitis and reproductive management strategies (e.g., dry cow therapy, hormonal synchronization technologies, heat detection methods, or both) used in the herd allow farm management to formulate decisions specific to the herd.

**Figure 1:**
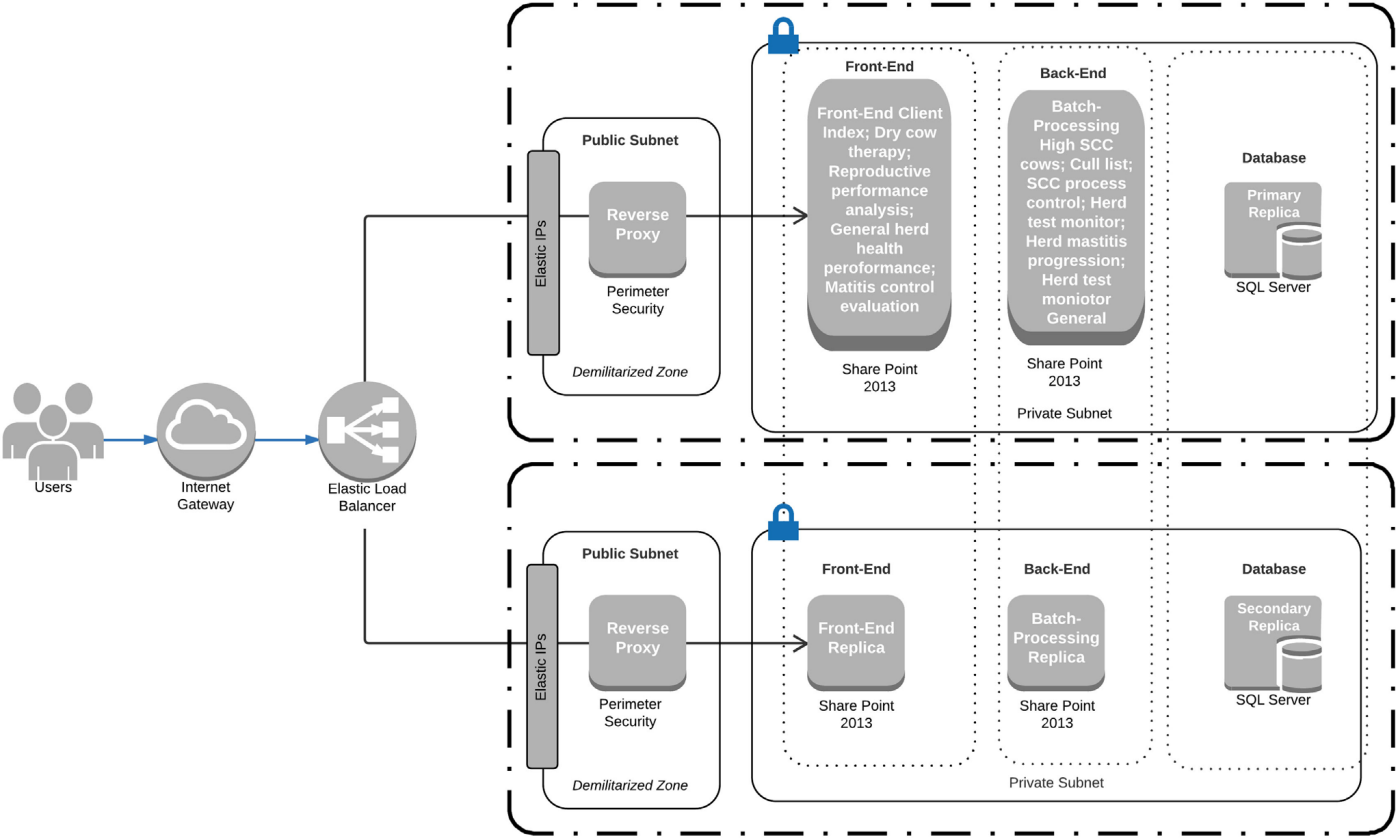
Network map for a web-based DSS*i*plus application designed to analyze herd tests and mating records and drug sales records from the New Zealand Livestock Improvement Corporation online database and VetLink^®^ accounting software, respectively. SCC, somatic cell count.

Figure 1 shows a network map for a web-based DSS*i*plus application designed to analyze cow-level and herd-level milk quantity and quality data, reproductive (insemination) events (LIC’s online database), and drug sales records (VetLink^®^ accounting software, Vetlinksql, Takapuna, Auckland, New Zealand) for a veterinary practice located on the North Island of New Zealand. On a daily basis, DSS*i*plus automatically acquired milk production, milk quality, and reproductive events data from two online database systems along with data from the in-house accounting software system.

Of-the-shelf, task automation software packages designed to perform automated tasks such as downloading data from online database systems are becoming more user friendly and more readily available. Open access statistical software packages are also available online. DSS*i*plus automatically acquires the data and then performs analyses of these data using open access statistical software (“*R”)* (7). Using R’s integrated suite of software packages facilitated all of the system’s data acquisition, data manipulation, and data analyses.

**Figure 2:**
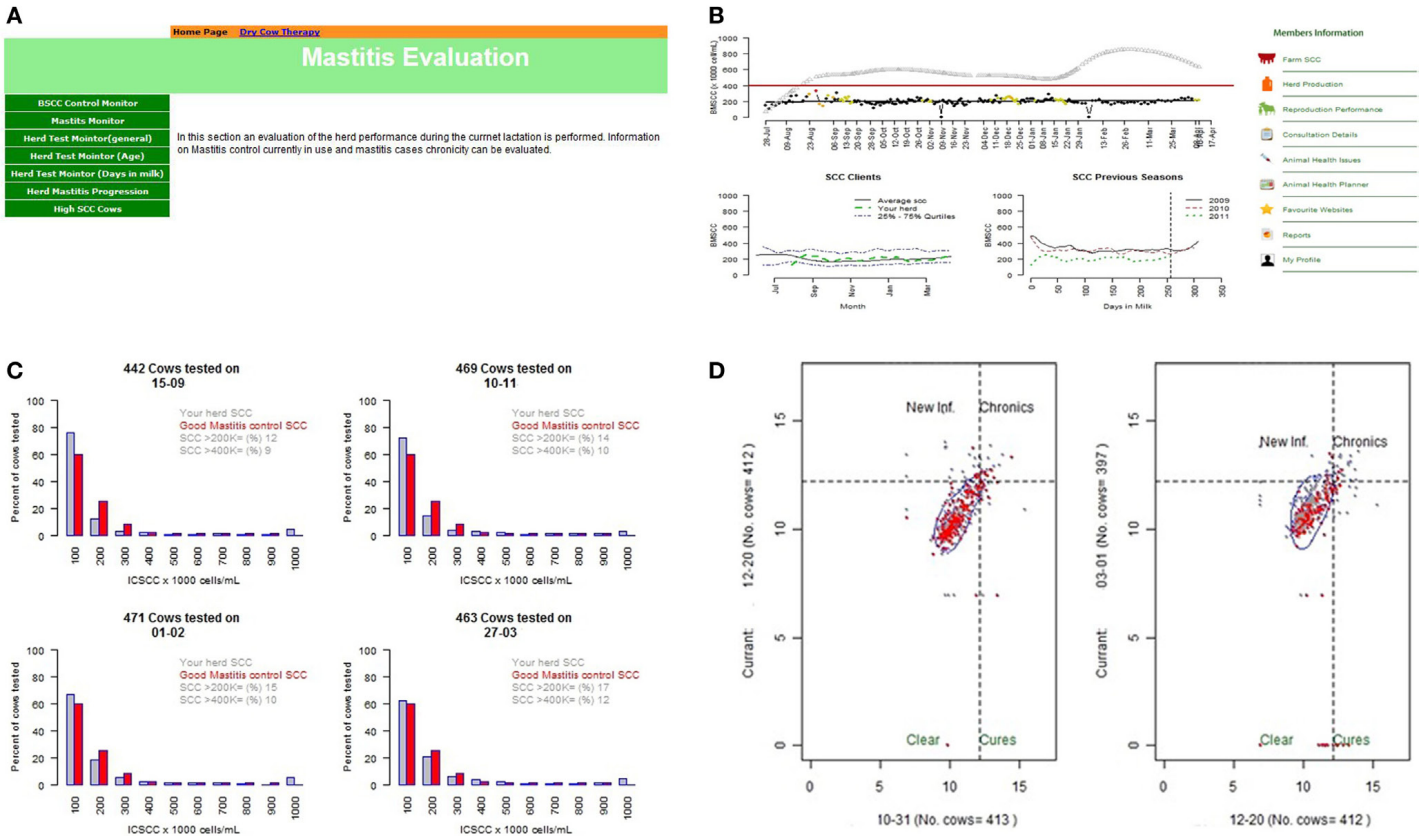
An example of descriptive outputs for the mastitis process control evaluation component **(A)** of the decision support system. **(B)** The Shewhart charts used by the DSS to monitor and benchmark milk volume-adjusted bulk tank milk somatic cell count and to identify unusual trends in milk quality of the dairy herd. **(C)** Pooled data from the top 25% performing herds used to monitor and set targets for individual cow somatic cell counts. **(D)** Log-log plots of individual cow somatic cell counts to monitor and evaluate changes in udder health status and estimate the efficacy of dry cow therapy.

Once the data were retrieved, outlier detection techniques remove outlier records and subject the cleaned data to various descriptive, analytical, and benchmarking analyses. For example, to assess the efficacy of a mastitis control program on a farm, quality control techniques (Shewhart charts) (8) were used to monitor somatic cell counts of bulk tank milk (adjusted for milk volume), identify unusual trends in the milk quality of dairy herds and to monitor mastitis control programs using lactating and dry cow therapy product sales as a proxy (Figure 2). DSS*i*plus utilized a series of descriptive and analytical data analyses techniques. Descriptive analyses (measures of central tendencies, measures of spread and frequency histograms) were used to visually inspect somatic cell count as a function of calendar day and milk volume. Analytical techniques (e.g., mixed effects modeling and smoothing splines implemented within *R*) were used to identify and remove outlier values, to implement Shewhart quality control techniques, and to benchmark herds based on the reproductive performance.

**Figure 3:**
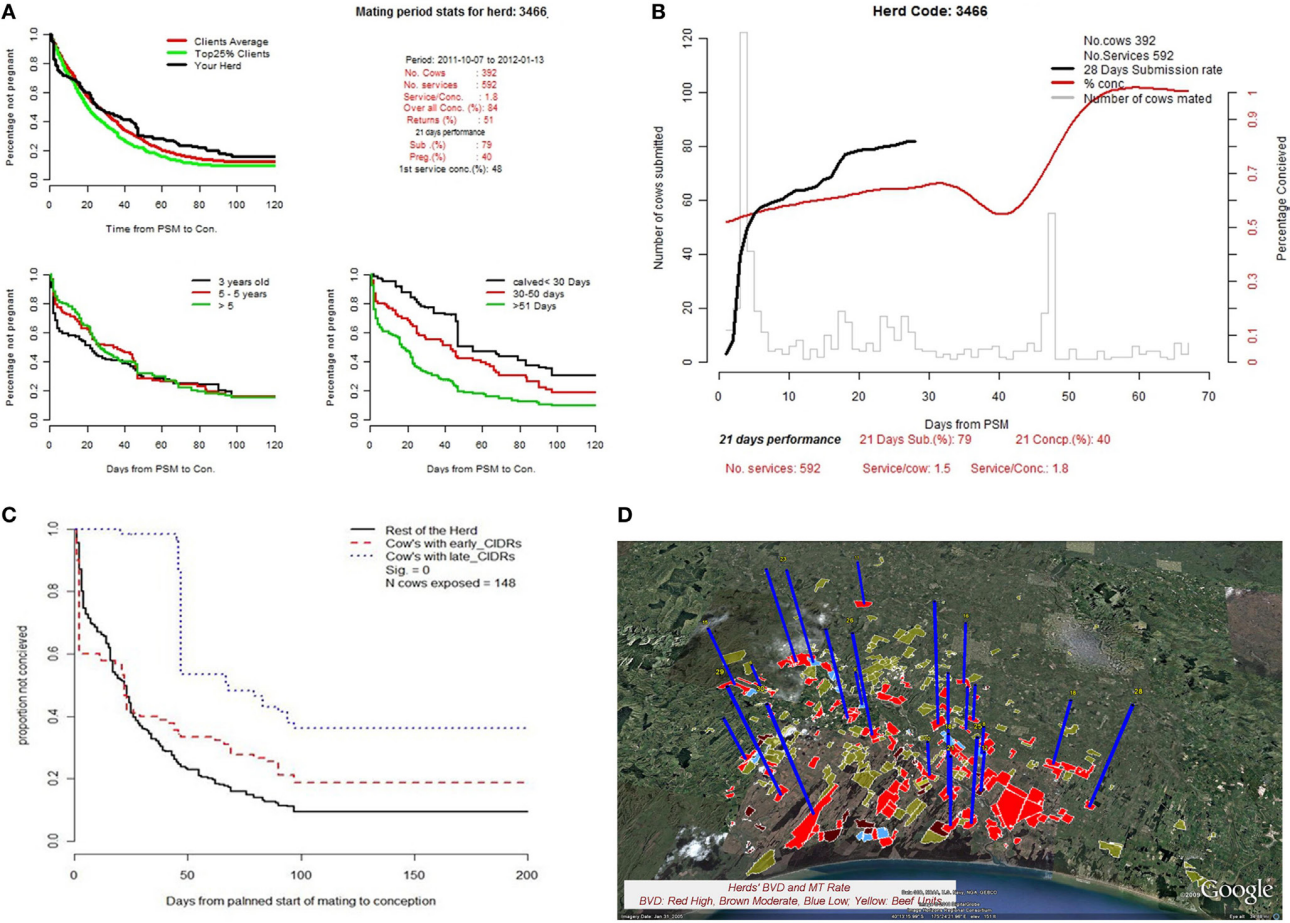
An example of analytical outputs for the reproductive performance evaluation component of the decision support system **(A–C)**. **(B)** Survival analyses techniques used to characterize and benchmark time-to-conception performance following the planned start of the mating period for each herd. **(C)** Efficacy of interventions aimed at improving herd reproductive performance using same methodology as for **(B)**. **(D)** Exploration of the crude association between a herd’s reproductive performance and factors believed to influence herd’s reproductive performance using pestivirus-positive herds. **(D)** The spatial distribution of pestivirus-positive herds is displayed as a function of herd reproductive performance.

DSS*i*plus also utilized log-log plots to analyze individual cow milk somatic cell count data as a means to characterize the efficiency of the mastitis treatment (lactating and dry cow therapy) and prevention (dry cow therapy) strategies used in the herd. The analyses track changes in udder health or mastitis severity through the lactation period. Crude statistical models were developed to estimate the number of clinical mastitis cases in a given herd based on the quantity of lactating and dry cow mastitis products sold to the client. This modeling process maximizes the utility of the accounting software by providing useful information for clinical decision-making. This procedure has allowed clients to be benchmarked as well as providing the opportunity to set goals for the next production season. Similar modeling techniques were used to analyze and benchmark client herds based on the herd’s reproductive performance (Figure 3). This process involved the identification of weaknesses in specific areas of the current operation of a client’s herd (calving interval, breeding program, animal health issues) that may be addressed to improve herd reproductive performance.

### DSSiplus Functionality and Interface Challenges

The main functionality, interface challenges that were identified in the development of the DSSiplus concept framework included data ownership, data acquisition and processing, and integration of DSS*i*plus with existing information systems currently used by veterinary advisors and choosing where to set the performance targets of a herd.

Several data sets are generated for dairy herds. For example, milk quality, insemination, and reproductive data are supplied to farmers on a fee-for-service basis. Farm managers and their advisors can access these data through third-party online information systems managed by the data custodians (DairyNZ and LIC). Standardized functions and procedures [application programming interface (API)] that would allow veterinary advisors to create specific applications and subroutines to access features of the operating information do not exist. This lack of standardized API prevented efficient data acquisition and processing. Therefore, data acquisition and interface protocols had to be updated regularly to ensure a reliable streaming of data from the custodians of the data to the DSS.

Dairy farm operation management software systems (for mastitis and somatic cell count monitoring and reproductive management) or decision support systems rely heavily on the quality of farm records and the quality of the data retrieved from custodians of the online database systems. “Cleaning-up” of the data is an essential step before any descriptive and analytical analyses can be conducted. Unfortunately, the functional capacity of the information systems used by the data custodians does not go beyond simple record keeping summaries. The data outlier detection protocols used in the development phase of the DSS identified a number of anomalies in the row data that could influence the accuracy of the record keeping summaries accessible to farm management. These include, but were not limited to, extreme and biologically implausible records (particularly for milk quantity and quality) and duplicate animal-level records. Moreover, the herd is a dynamic population. Therefore, appropriate statistical techniques need be used to account for the herd’s dynamic denominator data (i.e., the herd size at a given point in time) before any analytical procedures are applied on the data. Without appropriate data clean-up procedures and an accounting for the dynamic nature of the herd, simple summary statistics or measures of effect (e.g., proportions or rates) can be biased (overestimation or underestimation of effects) and imprecise. Bias reduction and enhancement of data precision are essential components of any system and critical to the transitioning from unstructured to structured, or semistructured, decision-making in dairy farm management (4, 5).

The concept of the performance targets underpins DSS*i*plus. Performance targets are created to optimize herd productivity in economic terms. The objective of the DSS is to define or monitor those targets that most closely relate to the economic efficiency of the herd (9, 10). A DSS should be able to benchmark the current reproductive performance of a herd (e.g., calving spread) or the somatic cell counts of bulk tank milk against that of previous production seasons and express the differences as a function of the herd’s average milk production per lactation or the average milk production per cow.

Setting performance targets can be based on experience (using pooled client data) or on a review of the relevant literature. However, it is critical that the targets be set as herd targets and not as individual cow targets, since the foundation of the whole approach is based on treating the herd as the unit of performance.

Therefore, the first task in setting performance targets is to examine that particular aspect of the production process in detail and decide upon one or more productivity indicators that will be used to represent a realistic economic target for the herd. An assumption inherent in this process is that achieving the productivity targets for the herd is synonymous with achieving the economic target. This is an essential first step in developing a DSS that will drive an evidence-based herd health program.

## Conclusion

Dairy farm managers and their advisors have access to an array of underutilized data sources. At a low cost to dairy producers and their advisors, an in-house DSS is capable of automatically acquiring milk and herd performance data. Retrieved data can be interrogated using a variety of open access statistical packages to identify trends or explore significant associations in the data. The transformed information enhances the abilities of herd health advisors’ to track and benchmark herd health and production performance against set performance targets and allows the allocation of resources to interventions targeting herd health and productivity based on the best available evidence.

## Author Contributions

JA conducted the work described in this paper and wrote the manuscript. JH and TO contributed to the concept and application of the described work and edited the manuscript.

## Conflict of Interest Statement

The authors declare that the research was conducted in the absence of any commercial or financial relationships that could be construed as a potential conflict of interest.
